# TCR-Signaling Events in Cellular Metabolism and Specialization

**DOI:** 10.3389/fimmu.2015.00292

**Published:** 2015-06-08

**Authors:** Danielle A. Chisolm, Amy S. Weinmann

**Affiliations:** ^1^Department of Microbiology, University of Alabama at Birmingham, Birmingham, AL, USA

**Keywords:** metabolism, glycolysis/gluconeogenesis, T cells, c-Myc, HIF-1α, Bcl-6, IRF4, T-bet

## Abstract

Engaging the T cell receptor (TCR) with peptide:MHC complexes initiates a cascade of signaling events that activates T cells in an antigen-specific manner. It is now clear that multiple inputs, including the strength of TCR signaling, co-stimulation, and the cytokine environment, impact T cell specialization decisions in the context of specific pathogenic encounters. Additionally, it is now appreciated that these same stimuli direct cellular metabolism programs. In this review, we will discuss how TCR-signaling events coordinate cellular metabolism and specialization gene programs in T cells.

The specialization of T cells is critical for controlling diverse pathogenic insults. Both CD4^+^ and CD8^+^ T cells have the capacity to differentiate into specialized effector and memory cells (1–3). Effector and memory T cells are responsible for fighting pathogens during an initial exposure or after a reencounter with the pathogen. In addition to the effector versus memory cell decision, CD4^+^ T cells also have the ability to differentiate into specialized effector subtypes such as T helper type 1 (Th1), Th2, Th17, T follicular helper (Tfh), and regulatory T (Treg) cells (4). These CD4^+^ T cell subtypes coordinate and regulate the immune response to deal with diverse types of pathogens. The importance of generating specific T cell subtypes in a context-dependent manner is highlighted by the pathogenic consequences that arise when there is an inappropriate balance between the specialized populations in a given setting. In particular, dysregulation of this process can cause autoimmune states or result in the inability to control an infectious agent (5, 6). Therefore, the molecular programs that define functionally distinct T cell subtypes must be precisely regulated in a context-specific manner to coordinate this intricate process.

Defining the series of molecular events that regulate T cell differentiation decisions has been a highly active research topic over the past several decades. The current research indicates that many diverse regulatory events contribute to the specialization decisions for T cells. These events are initiated at the time of pathogen encounter by T cell receptor (TCR) signaling, the stimulation of co-receptor complexes, and the cytokine environment (7–9). Precisely engaging these pathways coordinately regulate the gene expression programs that are necessary for specialization decisions. In this review, we will focus our discussion on how specific transcription factors translate TCR-signaling events into distinct metabolic gene expression programs to coordinate T cell specialization decisions.

## TCR-Signaling and T Cell Specialization

Each T cell expresses a unique TCR that is randomly generated during the process of VDJ recombination. This creates an expansive repertoire of antigen-specific T cells that are collectively capable of responding to diverse pathogenic insults (7). To date, many studies have focused on defining the signaling pathways induced by peptide–MHC interaction with the TCR (10). Importantly, these TCR-dependent signaling pathways modulate the expression and activities of key transcription factors (11–13). The transcription factors then effectively translate TCR signaling events into specialized gene expression programs.

Intriguingly, a series of studies suggest that an individual T cell displays some preference for the type of specialization program it initiates (e.g., effector versus memory; diverse CD4^+^ T cell subtypes) based in part upon characteristics related to TCR-signaling strength (14, 15). TCR signal strength encompasses both the affinity of the TCR for the peptide–MHC complex and the length of time that the peptide engages the TCR. This means that a unique TCR inherently has some preference for the type of specialization pathway that it selects (14, 15). However, the sequence of the peptide derived from the pathogen and how long the peptide–MHC complex engages the TCR will also influence the differentiation decision as well (16, 17). A new challenge for the field is now to define how graded TCR signal strength is translated through key transcription factors into diverse cellular specialization programs.

## TCR-Signaling Regulates the Transcription Factors that Influence T Cell Metabolism

**Figure 1:**
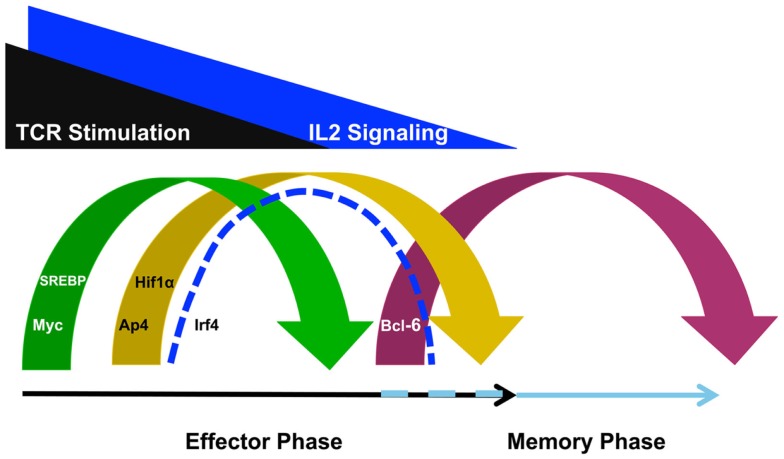
**T cell receptor-inducible transcription factors play non-redundant roles in defining the metabolic gene programs required for effector and memory cell potential**. TCR stimulation induces the expression of Myc to initiate a metabolic gene program that promotes effector cell specialization. TCR- and IL-2-signaling also induce transcription factors such as AP4, HIF1α, IRF4, and SREBP. These factors are required to maintain the glycolysis, glutaminolysis, and lipid biosynthesis pathways for maximum effector cell potential. By contrast, as TCR- and IL-2-signaling diminishes, Bcl-6 expression is upregulated to repress the effector cell metabolic program and transition the cell toward memory potential, possibly maintaining the program as well. Although the discussion in this review focused on the roles for these TCR- and IL-2-sensitive transcription factors in regulating metabolic gene programs, these factors also regulate other gene programs involved in cellular proliferation and survival as well.

It is becoming apparent that T cell specialization decisions are closely linked with changes in the metabolic programing of the cell. In particular, effector CD4^+^ and CD8^+^ T cells upregulate the glycolysis program, while memory T cells downregulate this program and instead rely on fatty acid oxidation (18, 19). Importantly, the selection of the predominant cellular metabolism pathways utilized in different T cell populations is in part controlled at the gene expression level. That is, the genes that encode components of the glycolysis, glutaminolysis, and lipid biosynthesis pathways are highly expressed in effector T cells whereas these same gene expression programs are inhibited in memory T cells (12, 13, 20, 21). In this context, it is striking to note that TCR-signaling events induce Myc, AP4, HIF1α, IRF4, and sterol regulatory-element binding protein (SREBP) family members (Figure 1), all of which are key transcription factors that regulate the metabolic gene expression program in T cells (11–13, 20, 22).

## TCR-Signaling Induces the Factors That Control Glycolysis and Glutaminolysis in T Cells

T cell receptor-signaling rapidly induces the expression of the transcription factor Myc (Figure 1). Myc expression rewires many aspects of the T cell gene expression program to activate genes encoding factors in metabolic pathways, such as glycolysis and glutaminolysis, as well as the gene pathways important for cell cycle progression (12). This Myc-dependent gene expression program is required for the initial burst of cellular proliferation that drives effector CD4^+^ and CD8^+^ T cell expansion. After the initial Myc-dependent events occur, TCR-signaling reinforces the effector cell program by inducing additional regulatory factors including AP4, HIF1α, and IRF4 (12, 20, 22, 23). It is thought that this second wave of TCR-inducible transcription factors take over the regulation of critical cellular processes from Myc (Figure 1). HIF1α and AP4 regulate the expression of genes that encode enzymes that are involved in glycolysis and glutaminolysis (20, 22, 24). Therefore, HIF1α and AP4 sustain the metabolic changes necessary for the effector potential of CD4^+^ and CD8^+^ T cells as Myc expression diminishes.

The transcription factor IRF4 appears to play a slightly different role in translating TCR-signaling into a gene program tailored for effector T cell specialization. In particular, several studies suggest that IRF4 is sensitive to the overall strength of TCR-signaling and gradations in IRF4 expression contribute to fine-tuning the metabolic program of T cells (11, 25, 26). The absence of IRF4 prevents the sustained expression of the metabolic gene programs that are required for effector cell specialization and IRF4-deficient CD8^+^ T cells fail to maintain their proliferative potential causing defective effector responses. Importantly, IRF4-dependent activities are more pronounced when the TCR is engaged by high-affinity antigens (11). Taken together, the current data have led to the speculation that IRF4-sensitive changes in the metabolic gene expression program translate the strength of TCR-signaling into functionally diverse effector T cell repertoires.

## TCR-Signaling Regulates the Factors Required for the Lipid Biosynthesis Gene Program

T cell receptor-signaling also induces the SREBPs (13). A great deal of the research, to date, regarding the programing of T cell metabolism has focused on defining the mechanisms that regulate the expression of the glycolysis and glutaminolysis pathways (18). However, other metabolic programing changes, including the activation of the lipid biosynthesis pathway, are required for robust proliferation and effector T cell differentiation (12, 13). The lipid biosynthesis or sterol pathway supports the rapid proliferation of T cells because lipids and cholesterol are required for the formation of new cellular membranes, as well as for assembling components of signal transduction pathways. Importantly, TCR-signaling induces *Srebf1* and *Srebf2*, the genes that encode SREBP1 and SREBP2, respectively (13). The SREBP family plays a role in inducing genes that encode numerous required components of the lipid biosynthesis pathway such as *Hmgcr*, *Acaca*, and *Fasn*. In the absence of SREBP activity, the lipid biosynthesis gene program is diminished in effector CD8^+^ T cells, resulting in severely impaired proliferation (13). Phenotypically, this causes a defect in effector CD8^+^ T cell responses and prevents the clearance of viral infections. Together, these data suggest that the TCR-dependent induction of SREBP activity is required for the activation of the lipid biosynthesis gene program and the development of effector T cell responses.

## Metabolic Transitions Associated with Memory T Cells

Many studies have defined the TCR-inducible regulatory factors that control the metabolic gene expression programs that promote effector T cell specialization. By contrast, the transcription factors that regulate the metabolic gene programs that promote memory T cell formation are much less defined (19, 27). Current data suggest that at least part of the memory T cell pool is derived from contracting effector T cells as antigen is cleared. There are at least two scenarios that might contribute to the mechanisms that regulate the metabolic gene program that promotes memory cell formation as T cells transition from effector to memory potential (Figure 1). The first possibility is that the glycolysis, glutaminolysis, and lipid biosynthesis programs are actively repressed as effector T cells transition into the memory phenotype. In support of this possibility, a recent study demonstrated that Bcl-6 actively represses the glycolysis pathway gene program when T cells are maintained in low IL-2 conditions (28). This is especially interesting to note because IL-2 becomes limiting with pathogen clearance at the time when the effector T cell population begins to contract and some cells are transitioning into memory potential. Together, these data suggest that Bcl-6 has the ability to actively repress the glycolysis pathway gene program during the effector to memory T cell transition to initiate a metabolic gene program compatible with memory T cell formation (28, 29).

The loss of key transcriptional activators is another mechanism that might contribute to changing the metabolic gene expression program between effector and memory T cells. The clearance of pathogen during the effector phase of the immune response will remove the antigen-specific stimulation of the TCR, first diminishing, and then ending, TCR-signaling. Therefore, as antigen is cleared and TCR-signaling wanes, the expression of Myc, HIF1α, AP4, IRF4, and SREBP expression will diminish (Figure 1). From a mechanistic perspective, the genes that encode components of the glycolysis, glutaminolysis, and lipid biosynthesis pathways will not be maintained at high-expression levels if the activators that induce their expression in T cells are not present. This means that the enhanced expression of the metabolic pathways that are actively engaged in effector T cells will likely not occur in the quiescent, long-lived memory cell population due to the absence of the activators Myc, HIF1α, AP4, IRF4, and SREBP. Thus, in the long-term steady state, continuous repression mechanisms will likely not be necessary to maintain the low level expression of the genes that encode components of these metabolic pathways in resting memory T cells.

## Integrating TCR- and Cytokine-Signaling in T Cells

Cytokine-signaling regulates the expression and activities of many transcription factors that are important in T cell metabolism and specialization decisions (3). The early cytokine environment is defined by the innate immune response to the pathogen. However, the predominant composition of the cytokine environment changes over the duration of the response, with later contributions originating from both innate and adaptive immune cells. The cytokine environment can control the expression and activity of the lineage-specifying transcription factors that influence cellular specialization at the onset of the immune response, as well as while the immune response develops and matures (3, 4, 30, 31). In CD4^+^ T cells, this translates into the development of specialized subtypes, such as Th1, Th2, Th17, Tfh, and Treg cells (4, 30).

In some cases, cytokine signaling modulates a similar set of regulatory factors to those induced by TCR. One cytokine that fits into this category is IL-2. IL-2-signaling reciprocally regulates the expression of HIF1α and Bcl-6 in CD4^+^ and CD8^+^ T cells (20, 28, 32, 33). Specifically, high IL-2 conditions promote HIF1α while inhibiting Bcl-6 expression, whereas by contrast, weak IL-2-signaling promotes the induction of Bcl-6 expression while inhibiting HIF1α (20, 28). Mechanistically, this sets up an antagonistic relationship between HIF1α and Bcl-6 in the IL-2-sensitive regulation of the glycolysis and glutaminolysis pathway gene expression programs in the context of effector versus memory cell formation (28).

Current research also suggests that many of the lineage-specifying factors required for CD4^+^ T cell specialization are also involved in regulating cellular metabolism. The Th1-lineage-specifying transcription factor T-bet plays a required role in Th1 specialization and also is important in the IL-2-dependent induction of the glycolysis pathway gene expression program in CD4^+^ Th1 and CD8^+^ T cells (28). In addition, Bcl-6, HIF1α, and IRF4 also have roles in regulating both T cell specialization and metabolism (11, 20, 24, 28). IRF4 plays a role in Th17 specialization and HIF1α is important for defining the specialization decision between Th17 and Treg cells (34–36). Bcl-6 is required for Tfh development and plays a role in memory cell specialization (3, 37). It will be interesting in future experiments to define whether the activities of other transcription factors important in the specialization of CD4^+^ T cells coordinate cellular metabolism as well.

## Conclusion and Future Directions

The transcription factors that are induced by TCR- and cytokine-signaling regulate both cellular specialization and metabolism, which suggest that these two processes are closely linked in T cells. In particular, effector cell specialization is associated with enhanced glycolysis, glutaminolysis, and lipid biosynthesis, whereas these same pathways are held in check in memory cell differentiation. Many elegant studies in CD8^+^ T cells have now defined the TCR-inducible transcription factors that regulate key metabolic pathways in effector T cell specialization, with the factors involved in memory cell formation still emerging. The current data suggest that both CD4^+^ and CD8^+^ T cells utilize similar transcriptional mechanisms to regulate the metabolic gene programs important for effector and memory cell specialization programs. Determining whether similar compositions of regulatory factors play roles in coupling characteristic metabolic pathways to states of specialization in different immune cell types is an important area for future research. Intriguingly, many of the transcription factors discussed in this review are also found in diverse immune cell types and their expression patterns in these populations appear to hint at similar functional roles related to effector and memory cell specialization (38–40).

Related to this topic, it will now be important to determine the identities of the TCR-independent signaling pathways that precisely control the activities of the factors discussed in this review in other cellular settings. Perhaps the responsiveness of these pathways to specific cytokines, such as IL-2, will be conserved in different cell populations. It is notable that many of the transcription factors that regulate cellular metabolism programs in effector T cells are highly expressed in cancer cells (41). Similarities between effector T cells and cancer cells have long been recognized in regard to their utilization of aerobic glycolysis and high rates of proliferation (19). Thus, it will be interesting to determine whether other immune cells that display similar proliferative characteristics utilize similar transcriptional regulation mechanisms to couple metabolic programs with highly proliferative cellular states.

Transcription factors are members of large families of proteins, with family members displaying both conserved and factor specific functions (6). Therefore, another area of research exploration will be to define the roles for related family members in different cellular settings. Recently, an intriguing connection emerged between the ZBTB transcription factor family and the repression of glycolysis. The ZBTB transcription factor Bcl-6 actively represses genes that encode components of the glycolysis pathway in T cells, while ZBTB7a represses a similar set of glycolytic target genes in MEF cells (28, 42). Importantly, ZBTB transcription factors are involved in cellular specialization decisions in diverse immune cell types, possibly suggesting that different members of this family might regulate similar gene expression pathways in different cellular settings (6). It will now be interesting to explore the role for other members of the transcription factor families discussed here in regulating metabolism gene programs in the context of diverse immune cell populations. In this regard, it will be informative to determine whether the activities associated with the TCR-inducible transcription factors in T cells are performed by the same or distinct family members in different cellular settings. Taken together, a vast amount of information has been uncovered to define how TCR-signaling is translated into cellular specialization programs and this knowledge is providing a wealth of insight into how the transcriptional programs that regulate metabolism are coupled to cellular specification decisions.

## Conflict of Interest Statement

The authors declare that the research was conducted in the absence of any commercial or financial relationships that could be construed as a potential conflict of interest.
